# A long postreproductive life span is a shared trait among genetically distinct killer whale populations

**DOI:** 10.1002/ece3.7756

**Published:** 2021-06-16

**Authors:** Mia Lybkær Kronborg Nielsen, Samuel Ellis, Jared R. Towers, Thomas Doniol‐Valcroze, Daniel W. Franks, Michael A. Cant, Michael N. Weiss, Rufus A. Johnstone, Kenneth C. Balcomb, David K. Ellifrit, Darren P. Croft

**Affiliations:** ^1^ Centre for Research in Animal Behaviour University of Exeter Exeter UK; ^2^ Pacific Biological Station Fisheries and Oceans Canada Vancouver BC Canada; ^3^ Department of Biology University of York York UK; ^4^ College of Life and Environmental Sciences University of Exeter Penryn UK; ^5^ Center for Whale Research Friday Harbor WA USA; ^6^ Department of Zoology University of Cambridge Cambridge UK

**Keywords:** kinship dynamics, postreproductive life span, menopause, *Orcinus orca*

## Abstract

The extended female postreproductive life span found in humans and some toothed whales remains an evolutionary puzzle. Theory predicts demographic patterns resulting in increased female relatedness with age (kinship dynamics) can select for a prolonged postreproductive life span due to the combined costs of intergenerational reproductive conflict and benefits of late‐life helping. Here, we test this prediction using >40 years of longitudinal demographic data from the sympatric yet genetically distinct killer whale ecotypes: resident and Bigg's killer whales. The female relatedness with age is predicted to increase in both ecotypes, but with a less steep increase in Bigg's due to their different social structure. Here, we show that there is a significant postreproductive life span in both ecotypes with >30% of adult female years being lived as postreproductive, supporting the general prediction that an increase in local relatedness with age predisposes the evolution of a postreproductive life span. Differences in the magnitude of kinship dynamics however did not influence the timing or duration of the postreproductive life span with females in both ecotypes terminating reproduction before their mid‐40s followed by an expected postreproductive period of about 20 years. Our results highlight the important role of kinship dynamics in the evolution of a long postreproductive life span in long‐lived mammals, while further implying that the timing of menopause may be a robust trait that is persistent despite substantial variation in demographic patterns among populations.

## Graphical abstract

The rare life history trait of a long postreproductive period in females have been linked to demographic patterns leading to an increased relatedness to the local group with age (kinship dynamics). Here, we show that two killer whale ecotypes that are genetically distinct and exhibit different demographic patterns as well as different strengths of expected kinship dynamics have evolved a long postreproductive period in females, supporting the prediction that an increased female local relatedness with age predisposes for the evolution of this life history trait. Moreover, this results indicates that the onset of the postreproductive period may be robust to substantial variation in demographic patterns among populations.

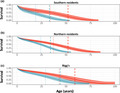

## 1 INTRODUCTION

The evolution of an extended female postreproductive life span is extremely rare in nature and is at present only known in five species of wild mammals (Croft et al., 2015; Ellis, Franks, Nattrass, Cant, Bradley, et al., 2017; Ellis et al., 2018). Outside of the prolonged postreproductive life span seen in humans the only other species of mammals in which females have evolved early cessation of reproduction are toothed whales: short‐finned pilot whales (*Globicephala macrorhynchus*), narwhals (*Monodon monoceros*), belugas (*Delphinapterus leucas*), and resident‐ecotype killer whales (*Orcinus orca*) (Ellis, Franks, Nattrass, Cant, Bradley, et al., 2017; Johnstone & Cant, 2010). Some evidence suggests that also the false killer whales (*Pseudorca crassidens*) have a substantial postreproductive period (Photopoulou et al., 2017). In resident‐ecotype killer whales, for example, adult females typically give birth to their last calf in their mid‐30s to early 40s followed by a postreproductive life span that may span many decades (Ellis, Franks, Nattrass, Cant, Bradley, et al., 2017). In the classical view of evolutionary theory, early termination of reproduction is not a beneficial trait (Hamilton, 1966; Williams, 1957) and understanding why and how the postreproductive life span has evolved remains a considerable challenge for evolutionary biology.

Adaptive explanations for the evolution of a long postreproductive life span have tended to focus on the inclusive fitness benefits of helping kin in late life (Kim et al., 2012; Nattrass et al., 2019; Sear & Mace, 2008). Females can gain inclusive fitness benefits in late life by ceasing reproduction and instead invest their energy in helping existing offspring survive and reproduce (“the mother hypothesis”) (Williams, 1957). Further, through behaviors that help increase the survival of grandchildren, such as providing ecological knowledge (Brent et al., 2015) or provisioning (Hawkes et al., 1997), postreproductive females can increase their inclusive fitness (“the grandmother hypothesis”) (Hawkes, 2004). In humans, grandmother benefits appear to be key for the evolution of a long postreproductive life span (Hawkes, 2004; Hawkes & Coxworth, 2013) and recent work in resident killer whales provides support for both the mother and grandmother hypothesis with the presence of both mothers and postreproductive grandmothers having a positive impact on the survival of their adult offspring and grandofffspring (Foster et al., 2012; Nattrass et al., 2019). However, the inclusive fitness benefits from helping are likely not on their own sufficient to explain the timing of menopause in both killer whales and humans (Hill & Hurtado, 1991) leading to the search for additional mechanisms that can contribute to the early termination of reproduction (McComb et al., 2001; Moss, 2001). Recent work has shown that kin‐selected costs, as well as benefits, are important for the evolution of extended postreproductive life spans (Cant & Johnstone, 2008; Johnstone & Cant, 2010).

Demographic patterns with either female‐biased dispersal and local mating or natal philopatry of both sexes and non‐local mating give rise to age‐specific changes in the relatedness of an individual to its group (kinship dynamics), in particular an increase in average female relatedness to other group members with age (Cant & Johnstone, 2008). In the case of resident killer whales, females are born into a social unit (“matriline”) consisting of their mother, siblings, and other more distant relatives (Bigg et al., 1990). As they age, their own sons replace more distantly related males in the matriline, increasing their average local relatedness to the group over time (Croft et al., 2017). This ultimately leads to an asymmetry in selection for helping and harming with age, which means that older females that are more related on average to the group are under stronger selection to help, while younger females are under stronger selection to harm (Johnstone & Cant, 2010). Thus, in competition for reproduction older females experience a larger inclusive fitness cost compared with younger females (Croft et al., 2017). The combination of such inclusive costs of intergenerational reproductive conflict and inclusive fitness benefits of helping kin are hypothesized to be key predictors for the evolution of a long postreproductive life span in mammals (Cant & Johnstone, 2008; Ellis, Franks, Nattrass, Cant, Bradley, et al., 2017; Johnstone & Cant, 2010).

Investigating kinship dynamics and age‐specific life history changes requires long‐term social and demographic data that captures most of the life span of animals. These data are therefore rare in long‐lived mammals. The long‐term data collected on different populations of killer whales in the coastal waters of the USA and Canada now extends over more than four decades, providing a unique opportunity to examine the link between kinship dynamics and life history evolution in a long‐lived marine mammal. In addition to the support for the mother and grandmother hypotheses in resident killer whales (Foster et al., 2012; Nattrass et al., 2019), there is strong support for the reproductive conflict hypothesis with offspring of older females that are born into conflict with offspring of a younger female having a 1.7 times higher mortality risk (Croft et al., 2017). However, it is still unknown whether these traits are shared between different populations of killer whales. Killer whales are among the most widely dispersed mammals on the planet and are found in all oceans (Baird et al., 1999; Ford, 2009). Lineages that differ morphologically (Baird & Stacey, 2011) and behaviourally (Riesch et al., 2012) and are genetically isolated (Morin et al., 2010) are referred to as ecotypes. Three killer whale ecotypes are sympatric in the northeast Pacific and among them are several populations of both resident and Bigg's killer whales (Barrett‐Lennard & Ellis, 2001; Parsons et al., 2013). In the waters off the west coast of North America the Northern and Southern populations of resident killer whales and the West Coast Transient populations of Bigg's killer whales can be found (Table 1; as well as a third, rarely encountered offshore ecotype not considered here) (Morin et al., 2010; Olesiuk et al., 2005; Parsons et al., 2013). Both populations of residents are specialist fish‐eaters with salmon making up almost all of their prey (Bigg et al., 1990; Olesiuk et al., 1990), whereas Bigg's killer whales are specialized in hunting marine mammals (Ford & Ellis, 1999). This differentiation in diet is reflected in the social behavior of the ecotypes with resident killer whales typically being observed traveling in larger social groups consisting of several maternal groups, compared with Bigg's killer whales (Towers et al., 2019, 2020). The mean group size of cohesive maternal groups however is similar for the two ecotypes (Table 1; Towers et al., 2020). In resident killer whales, there is almost no dispersal of males and limited dispersal of females from the maternal group. In contrast, there is a dispersal of both sexes from the maternal group of Bigg's killer whales (Baird & Whitehead, 2000; Ford & Ellis, 1999), which may be related to maintaining optimal group foraging size for predating on marine mammals (Baird & Dill, 1996; Krause & Ruxton, 2002).

**TABLE 1:** Overview of the populations of resident and Bigg's killer whales ecotypes occupying the waters off Washington State, USA, and British Columbia, Canada

Ecotype (Morin et al., 2010)	Genetic population (Parsons et al., 2013)	Prey specialization	Maternal group size Mean (range)	Social structure
Resident	Southern residents	Fish (mainly salmon) (Ford & Ellis, 2006)	4.33 (1–16) (Center for Whale Research)	Natal philopatry of males and females, non‐local mating (Bigg et al., 1987) (but see Ford et al. (2018))
	Northern residents	Fish (mainly salmon) (Ford & Ellis, 2006)	6.5 (1–19) (Towers et al., 2020)	Natal philopatry of males and some females, non‐local mating (Barrett‐Lennard & Ellis, 2001; Bigg et al., 1987)
Bigg's	West Coast Transients (WCT) (Sharpe et al., 2019)	Marine mammals (mainly pinnipeds and small cetaceans) (Ford & Ellis, 1999)	3.9 (1–8) (Towers et al., 2019)	Some dispersal of males and females from natal group, non‐local mating inferred (Baird & Whitehead, 2000; Ford & Ellis, 1999)

**FIGURE 1:**
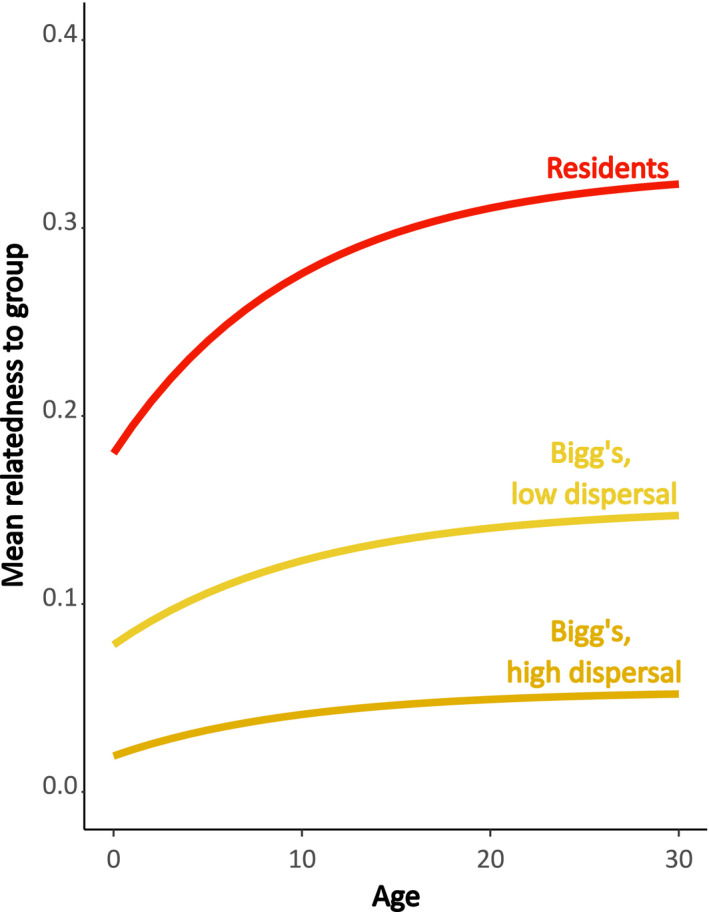
Predicted age‐specific mean relatedness to the group in female resident and two hypothesized Bigg's killer whales with different mating and dispersal patterns. Residents have bisexual philopatry with out‐group mating and Bigg's are shown here under two different scenarios of low degree of dispersal and high degree of dispersal, both with out‐group mating. Calculations are based on the theoretical models from Johnstone and Cant (2010). The model includes information about 4 parameters: the proportion of females that disperse (*d*) from their maternal group, the proportion of mattings that occur within the maternal group (*m*), the expected adult life span of females (µ), and maternal group size (*n*). The values used to estimate Bigg's female kinship dynamics is based on current knowledge on their dispersal and mating pattern (Towers et al., 2019). Two scenarios were chosen for Bigg's to illustrate a range for the kinship patterns, as the degree of dispersal and mating has yet to be quantified in the Bigg's ecotype. Resident parameters for bisexual philopatry and out‐group mating: *d* = 0, *m* = 0.01, µ = 50, *n* = 4. Bigg's parameters for low dispersal of both males and females and out‐group mating: *d* = 0.5, *m* = 0.2, µ = 38, *n* = 4. Bigg's parameters for high dispersal of both males and females and out‐group mating: *d* = 0.8, *m* = 0.2, µ = 38, *n* = 4

The difference in demography observed between the resident and Bigg's ecotypes is predicted to generate different patterns of kinship dynamics (age‐specific changes in relatedness) which can be illustrated using the theoretical model presented by Johnstone and Cant (2010). Previous theoretical work on kinship dynamics in resident killer whales predicted an increase in local relatedness with female age (Johnstone & Cant, 2010) which has been confirmed using individual‐based demographic and social data from resident killer whales (Croft et al., 2017). Here, we use this established modeling framework to predict the patterns of age‐related changes in kinship for female Bigg's killer whales allowing us to directly compare the predicted kinship dynamics between the resident and Bigg's ecotype. Using this approach, it is predicted that female local relatedness will increase with age for both killer whale ecotypes, albeit with a weaker relationship under the demographic conditions of Bigg's killer whales (Figure 1; Johnstone & Cant, 2010). This increase in female local relatedness is opposite to the pattern observed in most mammals (male dispersal and local mating), and it predicts that there will be selection for a postreproductive female life span in both killer whale ecotypes. We hypothesize however that the difference in the strength of the kinship dynamics will lead to a lower potential for inclusive fitness benefits from helping kin in late life and inclusive fitness costs of reproductive conflict with younger females for Bigg's females (Croft et al., 2017). Given the predicted differences in kinship dynamics, and assuming the costs and benefits of reproduction with age are equal and that these do not change with changing dispersal, we predict that selection for a postreproductive life span will be weaker in Bigg's killer whales compared with resident killer whales (Johnstone & Cant, 2010) and we further hypothesize that this will be reflected by an older age at last reproduction, and a shorter postreproductive life span in female Bigg's killer whales.

Here, we test for the presence of a postreproductive period in Bigg's killer whales and compare female postreproductive life span between the sympatric resident and Bigg's killer whale ecotypes. Using over 40 years of individual‐based demographic data, we model survival trajectories using a Bayesian hierarchical framework (Colchero & Clark, 2012) and calculate the postreproductive representation (PrR) (Levitis & Lackey, 2011) to compare the presence of a long postreproductive life span in both resident and Bigg's killer whale ecotypes, as well as the timing of a potential postreproductive life span. We show that females in both ecotypes have a prolonged postreproductive life span. However, in contrast to our predictions, the timing and duration of the female postreproductive life span did not appear to differ between ecotypes.

## 2 METHODS

Here, we use postreproductive representation (PrR) as a measure for postreproductive life span, which is measured as the proportion of adult years being lived as postreproductive (Levitis & Lackey, 2011), and in the context of this study, it is the proportion of female years which are lived postreproductively. While a decline in fertility with age is a general trait among mammals (Packer et al., 1998), the long postreproductive periods, often spanning more than a decade, observed in humans and some toothed whales is a rare trait. Reports of postreproductive life span in other species often reflect individual variation in senescence, rather than a general trait at the population level are calculated for populations living under artificial conditions often with reduced mortality risk (Cohen, 2004; Croft et al., 2015; Levitis et al., 2013; Packer et al., 1992). A significant advantage of the PrR measure is that it is a population‐level measure that is directly comparable between species or populations with different lengths of life span as it is the number of female years lived postreproductively out of all years lived by females in a given population, while PrR also allows for a test of whether the postreproductive life span is significantly larger than what is expected by chance (Levitis & Lackey, 2011).

### 2.1 Data type and collection

Long‐term photo‐identification data have been collected on three killer whale populations with overlapping geographical ranges in the waters off Washington State, the state of Alaska, USA, and British Columbia, Canada: Northern resident, Southern resident, and Bigg's killer whales (Towers et al., 2019) (Table 2). Annual photo‐identification studies began in 1972 for Bigg's, 1973 for Northern residents, and 1976 for Southern residents. Data collection were boat‐based and during each encounter identification photographs of dorsal fins and saddle patch were obtained from the left side of each whale (Bigg et al., 1976, 1987). Sex was determined based on the pigmentation of the ventral side of genital or mammary slits, the presence of neonates, or the size and shape of the dorsal fin of adults (Towers et al., 2019).

**TABLE 2:** Overall summary of killer whale photo‐ID datasets

	Southern residents	Northern residents	Bigg's
Study start	1976	1973	1972
Collected by	Center for Whale Research	Fisheries and Oceans Canada	Fisheries and Oceans Canada
Individuals in dataset	207	564	506
Females (adult^a^)	94 (67)	193 (159)	180 (145)
Males (adult^a^)	86 (45)	170 (124)	121 (82)
Unknown sex	27	201	205
Known year of birth	128	433	351
Known year of death	124	238	126
Known year of birth and death	59	131	70
Adult sex ratio^b^	0.40	0.44	0.36

^a^ Adult males and females (individuals of age 13 or over in the datasets).

^b^ Adult sex ratio is calculated as the fraction of all adult individuals that are male.

The Center for Whale Research (www.whaleresearch.com) collected the long‐term dataset for Southern residents. With all alive members of this population being observed each year of the study, this provides a near‐complete life history for the population including information on survival for males and females and reproduction for females. Fisheries and Oceans Canada collected the long‐term data on both Bigg's and Northern residents, which includes the survival for most individuals and reproduction for females. However, a multi‐year lack of encounters with some matrilineal groups generated uncertainty for year of death of some individuals, especially for Bigg's killer whales (Table 2). We therefore limited our analysis to a subset of the most commonly documented individuals in this population (Towers et al., 2019). We used the photo‐identification data from all populations in the format of a capture–mark–recapture sighting matrix, that is, absence/presence for each individual during each year. The high overall annual recapture probabilities provide reliable long‐term data on these populations (Bigg et al., 1990; Towers et al., 2019, 2020).

In all three datasets, year of birth was only included for animals that were born after the start of the given study, otherwise year of birth was zero, indicating birth year as unknown (Table 2). In Southern residents, newborn individuals were observed within their first year of life. In Northern residents and Bigg's, if an individual was not observed within the first year of life, year of birth was estimated based on body size of the individual when it was first observed relative to calves of known ages. This method is reliable for calves observed within the first three years of life with a precision of ±1 year (Olesiuk et al., 2005; Towers et al., 2019). Uncertainties of the birth year >±1 year were classified as unknown for the purposes of this study. For all populations, year of death of an individual was determined either directly from strandings or based on an individual missing from its matrilineal group on a single (individual less than 3 years old) or on several (individual more than 3 years old) occasions (Towers et al., 2019). If year of birth and/or death were unknown for a given individual, these values were assigned a zero and would be estimated by the model (see Table 2 for number of individuals with known birth and death year).

### 2.2 Estimating age‐specific survival

As killer whales are a long‐lived species, a common feature of the observation data from all three populations is that some individuals were born before the studies started (i.e., left truncated) and some were still alive at the end of the study (i.e., right‐censored). This gives rise to uncertainties regarding some birth and death years in the datasets. Further, some individuals have gaps in their sighting history of more than 1 year likely due to their preference for waters beyond the core study areas. This introduces uncertainty in the recapture probability and year of death of these individuals. To account for the uncertainties and missing observations in the data, we used a Bayesian hierarchical framework to estimate the age‐specific survival and mortality for all three populations (Colchero & Clark, 2012). This framework estimates the unknowns and uncertainties as latent variables (i.e., variables to be estimated) and combines this with flexible parametric mortality functions to predict the age‐specific survival of the three killer whale populations (Colchero et al., 2012). Although the data on Southern residents are near‐complete, we have used the same approach on all datasets to ensure the results are directly comparable. Given that males and females likely have different mortality trajectories with males often having a higher mortality rate compared with females (Lemaître et al., 2020; Olesiuk et al., 1990), the sex of individuals was included as a covariate in the analyses.

We fitted 10 different mortality and survival models to each of the three datasets using the BaSTA package (Colchero et al., 2012) in R version 3.6.2 (R Core Team, 2019). The different functions tested either describes a constant mortality that is independent of age (Exponential), an exponential increase in mortality with age (Gompertz) (Gompertz, 1825), an increase or decrease in mortality as a power function of age (Weibull) (Pinder et al., 1978), or an initial exponential increase in mortality that plateaus after a given age (Logistic) (Vaupel et al., 1979). Adding a shape term to the mortality function allows the mortality to have an initial decline from birth (bathtub shape) (Siler, 1979) or an added constant mortality rate that is independent of age (Makeham) (Makeham, 1867). We ran the models for four Markov chain Monte Carlo (MCMC) chains to evaluate convergence of the model. The final model specifications, where convergence for all model types had been reached, were: four MCMC chains, 1,000,000 iterations, 200,001 burn‐in, and 10,001 thinning (Gelman et al., 2014). We visually inspected convergence in the parameter traces for the four chains, to ensure that all chains had mixed properly. Further, convergence was assessed formally for each model on the basis of the scale reduction ($\hat{R}$), where $\hat{R}$ < 1.1 indicates convergence (Colchero et al., 2012; Kruschke, 2014).

### 2.3 Testing the fit of the model

We used the deviance information criterion (DIC) to evaluate the model fit and predictive power of the different models, a measure analogous to Akaike's information criterion (AIC) (Spiegelhalter et al., 2002) (but see Spiegelhalter et al. (2014)). The importance of including the categorical covariate of sex was investigated using the Kullback–Leibler discrepancy, which is informed by the overlap of the posterior distribution of parameter estimates (Kullback & Leibler, 1951; Larson et al., 2016; McCulloch, 1989). For data including only individuals of known sex, the Gompertz model with a bathtub shape described the mortality and survival trajectory well for all three populations, as the second best fit for Southern and Northern residents and the best fit for Bigg's (Table 3). Mathematically, the Gompertz bathtub model consists of three elements.

$$\mu \;\left(x\right)=e^{a_{0}‐a_{1}x}+c+\exp\;\left(b_{0}+b_{1}x\right)$$

where *a_0_* and *a_1_* define the initial decline in mortality from birth, *c* defines the mortality through the adult stage and *b_0_* and *b_1_* define the exponential increase in mortality at the senescent stage. This model therefore offers great flexibility in the age of onset of aging as well as changes in the rate of aging throughout the life span (Colchero & Clark, 2012; Colchero et al., 2012; Siler, 1979) and we use this model for quantifying the postreproductive life span for all three populations to best ensure direct comparison between the three populations. Given the lack of data on older whales and the related uncertainty around ages at death, we included weakly informative prior distributions that allowed the models to explore the parameter space while reflecting a plausible range of resulting values for survival. These prior distributions were informed using known life history traits for the populations (Olesiuk et al., 2005). For the final model (Gompertz with bathtub shape), we ran the model with the following prior means (and standard deviations): *a*_0_ = −3 (σ = 1), *a*_1_ = 0.2 (σ = 0.05), *c* = 0.001 (σ = 0.001), *b*_0_ = −4 (σ = 1), and *b*_1_ = 0.05 (σ = 0.01). All prior distributions were normally distributed and truncated at 0, except for *a*_0_ and *b*_0_. Further, recapture probabilities for all three populations were assumed to be time‐dependent as there are variations in observation efforts across the study periods. This time dependency of recapture probabilities was therefore included in the model, as it allows for variation of the parameter estimates for each time period (10‐year period).

**TABLE 3:** Deviance information criterion (DIC) for the three models with the best fit for the three different populations with input data of individuals with known sex

Southern residents			Northern residents			Bigg's		
Model	Shape	DIC	Model	Shape	DIC	Model	Shape	DIC
Gompertz	Makeham	3,464	Gompertz	Simple	6,811	Gompertz	Bathtub	5,645
Gompertz	Bathtub	3,506	Gompertz	Bathtub	6,854	Gompertz	Simple	5,745
Weibull	Makeham	3,543	Logistic	Simple	6,981	Logistic	Makeham	6,003

### 2.4 Permutations of individuals with unknown sex

The sex of a substantial portion of individuals was not determined in the Northern resident and Bigg's populations (Table 2). These individuals were all under 15 years. In mammals, mortality is often higher in early life, and by excluding juvenile individuals from the analyses, we are likely underestimating such early‐life mortality. Instead of including them as a third category of “unknown sex”, which would—in essence—calculate the mortality trajectory of an artificial short‐lived sex, we used a permutation approach to assign a sex to these individuals randomly. This way we are able to include the early‐life mortality into the full age‐specific mortality trajectory. We implemented the same Bayesian hierarchical model that was the best overall fit for individuals of known sex, the Gompertz bathtub model, which was confirmed by a trial run on a set of random datasets as the best fit for the full data. Although only 21 individuals were of unknown sex in the Southern residents, we also ran the same number of permutations on this population for comparability. We ran 1,000 permutations, where sex was randomly assigned to individuals of unknown sex for each permutation. Both populations have a female‐biased adult sex ratio (Table 2), and we assumed a sex ratio of 1:1 at birth. We used the permuted output to calculate the variation in postreproductive representation and to compare mortality patterns between all three populations. As the estimates of maximum life span are often unreliable (Moorad et al., 2012; Ronget & Gaillard, 2020), we compare life span using average (expected remaining life span at birth) and 90% (the age where 90% of individuals in a population has died) life span (Moorad et al., 2012; Ronget & Gaillard, 2020).

### 2.5 Calculating postreproductive representation

**FIGURE 2:**
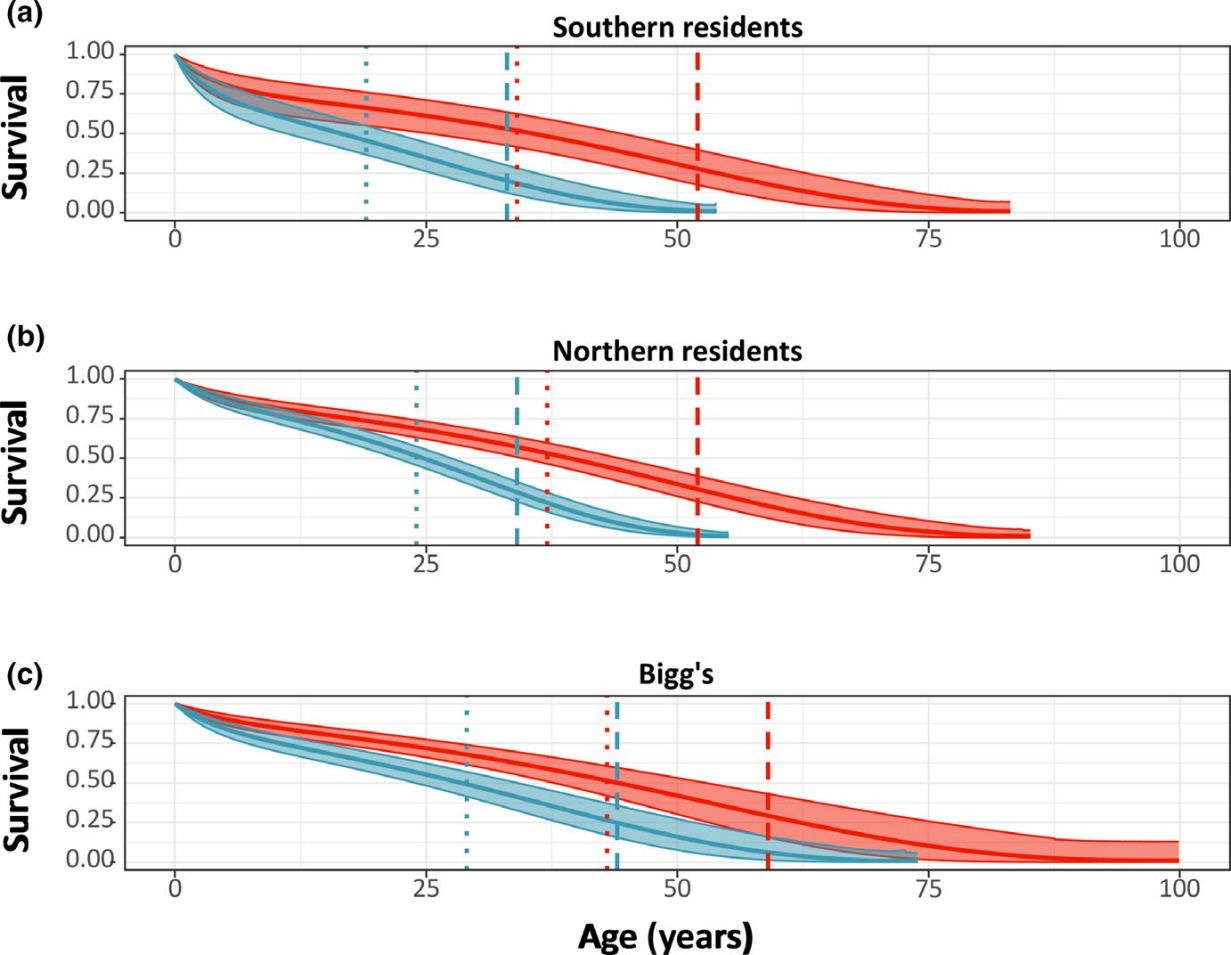
Average of the age‐specific survival trajectory for male (blue) and female (red) from all permutations with sex assigned randomly to individuals of unknown sex. Bold lines are median survival, and shaded areas represent median 95% credible interval. Average life span (dotted) and 90% life span (dashed) are indicated with vertical lines for males (blue) and females (red)

To calculate PrR, two data series were obtained, the *l*_x_ and *m*_x_ series. The first series, *l*_x_, is the probability of survival to a given age *x*, obtained from the survival model output (Figure 2). The second, *m*_x_, is calculated as the number of offspring born to females of a given age divided by the number of females that were alive at that age. We included all females with a known or estimated year of birth in our calculation of m_x_. Maternities were assigned from observations of mother‐calf interactions following the first observation of the calf (Ford & Ellis, 1999; Towers et al., 2019). Only calves born within the study period were used to estimate age‐specific fecundity. Females of a given age were classified each year as either (a) having given birth, (b) not having given birth, or (c) having an unknown birth status. The latter classification was used when a female was not observed in a given year and the presence or absence of a new calf could not be confirmed. We only included females of a given age with a known birth status (1 or 2) to derive m_x_. The proportion of females that had a known status of birth was 78% in Northern residents, 70% in Bigg's killer whales, and 93% in Southern residents. The proportion with unknown birth status did not systematically vary by age in any of the three populations.

Mathematically, PrR is based on *l*_X_ and *e*_x_, where *e*_x_ is the life expectancy at age *x*. A multiplication of these terms gives *T*_X_ (the total individual years lived after age *x*). PrR is then calculated from *T*_x_ at age *B* and age *M*, which are the ages, where 5% and 95% of female fecundity has been realized (inferred from *m*_x_). Thus, the formula for calculating PrR is:

$$\text{PrR}=\frac{T_{M}}{T_{B}}=\frac{l_{M}}{l_{B}}*\frac{e_{M}}{e_{B}}.$$

The input was a lifetable consisting of *l*_x_ (from the basta model output as this allows for the inclusion of both right‐censored and left‐truncated data, as well as individuals with missing data) and *m*_x_ (calculated from the observational data). To test the statistical significance of the PrR value, it was tested against the null hypothesis that survivorship and fecundity declines with the same rate, which would lead to PrR = 0. We simulated 9,999 populations of 1,000 individuals, where this null hypothesis was true and compared each of these null populations with each permutation of the observed population. The simulated null populations were generated based on the demographic parameters of the given killer whale population. The p‐value was obtained by evaluating how many of these simulated populations had a PrR greater than or equal to the PrR values obtained from the observed populations, with the number of samples included in both the numerator and denominator (Ellis, Franks, Nattrass, Cant, Bradley, et al., 2017; Ruxton & Neuhäuser, 2013).

## 3 RESULTS

Overall, 198 Southern resident, 568 Northern resident, and 510 Bigg's killer whales were observed in these populations over the >40 year period of observations (Table 1) and included in the analysis. The adult sex ratio for all datasets was female‐biased (Table 2). There was a substantial number of individuals, where the sex had not been determined with 202 in Northern residents and 209 in Bigg's killer whales (Table 2).

### 3.1 Age‐specific survival

The model (Gompertz with bathtub shape) reveals a clear age‐ and sex‐specific pattern of survival for all three populations (Figure 2) that have similar average and 90% life span (Table 4). We assessed the fit of the model by plotting the estimated survival probability together with the observed survival probability of individuals of known age (Figure S1).

**TABLE 4:** Average life span (the expected life span at birth) and the 90% life span (the age when 90% of the life span has been realized). Values are presented as median ± *SD* derived from the median and credible interval (in parentheses) from the 1,000 model permutations for each population

	Average life span	90% life span
Southern residents		
Females	34 ± 1.4 years (CI: 27 ± 1.3–42 ± 1.4 years)	52 ± 0.5 years (CI: 47 ± 0.4–58 ± 0.7 years)
Males	19 ± 0.5 years (CI: 15 ± 0.5–23 ± 0.6 years)	33 ± 0.4 years (CI: 29 ± 0.1–37 ± 0.4 years)
Northern residents		
Females	37 ± 0.7 years (CI: 33 ± 0.8–43 ± 0.9 years)	52 ± 0.5 years (CI: 48 ± 0.5–58 ± 0.9 years)
Males	24 ± 0.4 years (CI: 22 ± 0.5–27 ± 0.5)	34 ± 0.3 years (CI: 32 ± 0.4–37 ± 0.5 years)
Bigg's		
Females	43 ± 11 years (CI: 35 ± 1.0–51 ± 2.0 years)	59 ± 1.5 years (CI: 50 ± 1.2–69 ± 2.9)
Males	29 ± 0.7 years (CI: 24 ± 0.6–34 ± 1.0 years)	44 ± 0.7 years (CI: 38 ± 0.7–50 ± 1.3 years)

### 3.2 Postreproductive representation

**FIGURE 3:**
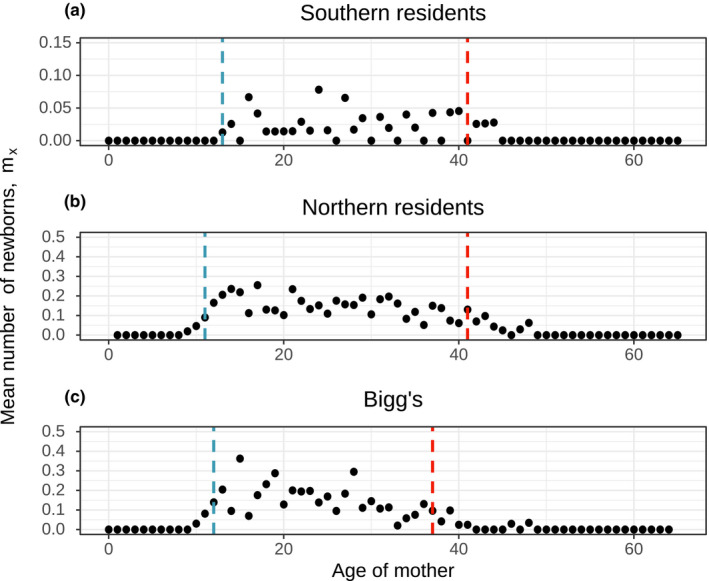
Age‐specific fertility, *m*_x_, for Southern resident, Northern resident, and Bigg's killer whales. Plotted values are the number of observed offspring born to females of age x divided by the number of observed females at risk of reproduction at age *x* (points), and dashed lines indicate age *B* (5% of fertility has occurred; blue) and age *M* (95% of fertility has occurred; red). Note the difference in scale on the *y*‐axis of *A*

**FIGURE 4:**
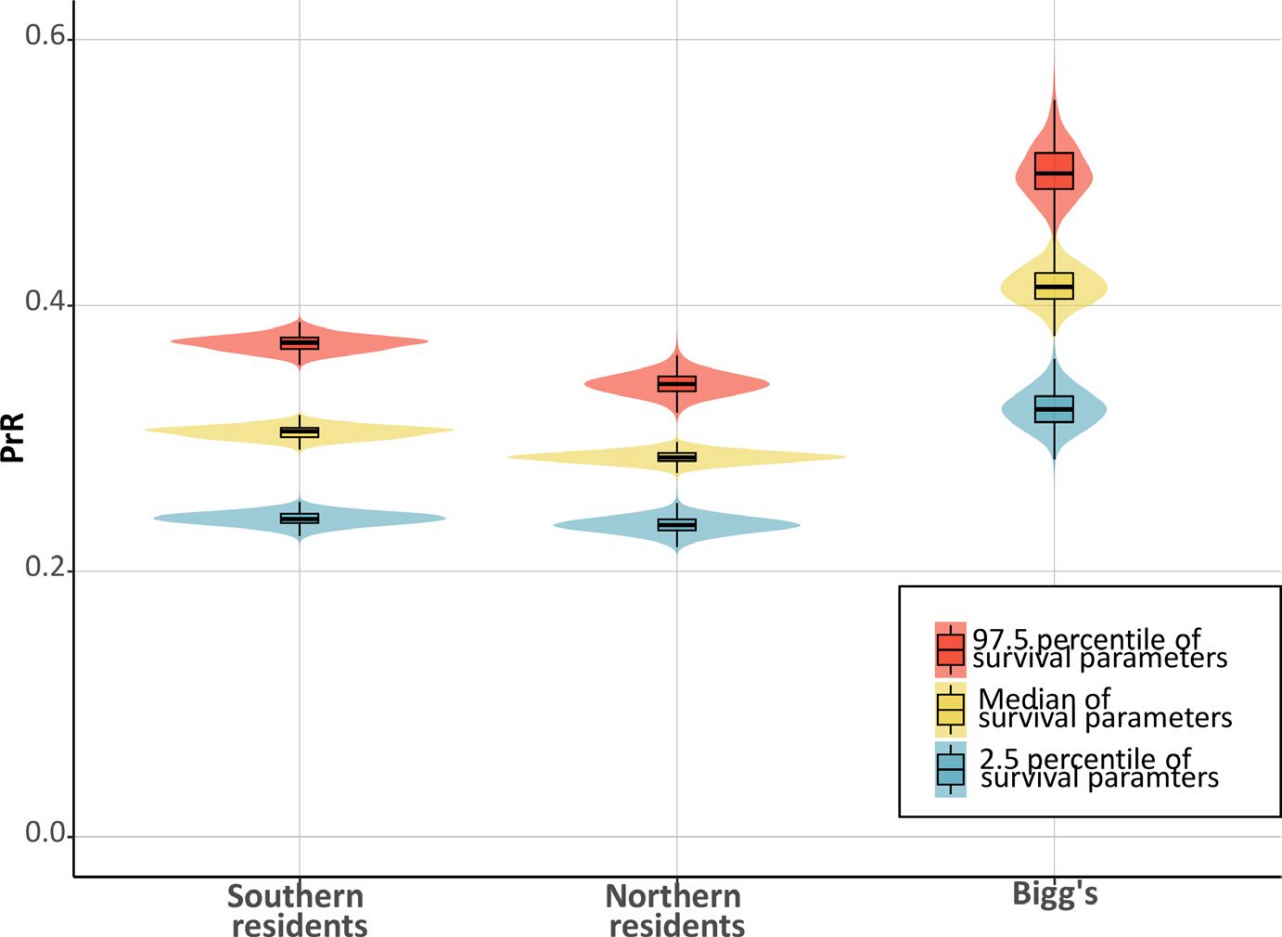
Estimated postreproductive representation (PrR) values for Southern resident, Northern resident, and Bigg's killer whales. The three violin plots for each population represent the kernel probability density of the PrR values calculated from the median (yellow) and the 95% credible interval (blue and red) survival trajectories (1,000 permutations for each population) with boxplots overlain to illustrate the median, quartiles, and whisker reaching up to 1.5*interquartile range. PrR was significantly higher than 0 for all permutations for all three populations (*p* < 0.05)

All three populations had a postreproductive representation that was significantly greater than zero (*p* < 0.05), with estimated PrR values above 0.3 for all three populations (Figure 4). Female fecundity had an initial increase at ~12 years, and 95% of fecundity had occurred at age 37–41 (Table 5; Figure 3). For all three populations, median expected female life span as postreproductive was >20 years (Table 5) and the probability of surviving until age *M* was median >40%.

**TABLE 5:** Overview of female fecundity and female expected additional life span for the resident and Bigg's killer whale populations at age *B* and age *M*, the ages at which 5% and 95% of female fecundity has occurred, respectively

	Age *B* (5% fecundity)	Additional years of life expected at age *B*	Age *M* (95% fecundity)	Additional years of life expected at age *M*
Southern residents	13^a^	39 ± 0.5^b^	41^a^	22 ± 0.4^b^
Northern residents	11^a^	39 ± 1^b^	41^a^	21 ± 1^b^
Bigg's	12^a^	46 ± 2^b^	37^a^	32 ± 2^b^

^a^ Fecundity (including age *B* and age *M*) is calculated from the number of births to females of a given age out of the number of alive females of that age.

^b^ Median expected life span (median ± *SD*) based on age‐specific survival from permutated models.

## 4 DISCUSSION

We show that three sympatric killer whale populations have comparably long postreproductive female life spans. With a median of more than 30% of all adult female years in all three populations being lived by postreproductive females, it is a substantial life history stage (Figure 4). This is the first evidence showing a long postreproductive period as a shared trait among several genetically distinct killer whale populations, suggesting that it could have evolved in a common ancestor to current killer whales and that it might be present in other discrete populations. Indeed, phylogenomic analysis suggests that the divergence between the lineages leading to resident and Bigg's ecotypes is the earliest among extant killer whales occurring over 350,000 years ago (Morin et al., 2015). If the shared presence of a postreproductive life span in both residents and Bigg's is due to a shared ancestral trait, this suggests that a postreproductive life span is an ancestral state of all extant killer whale ecotypes.

From the survival model output, the survival patterns are similar for all three killer whale populations, with females expected to live both substantially longer than males and to ages of >50 years (Figure 2). Interestingly, the model predicts that male Bigg's killer whales may have a longer expected life span compared with resident males (Figure 2), a pattern that could be influenced by the differences in their environments, both social (Ellis, Franks, Nattrass, Cant, Weiss, et al., 2017; Lemaître et al., 2020) and ecological (i.e., prey availability (Ford et al., 2010; Shields et al., 2018)). However, this could also be an effect of male dispersal in Bigg's, resulting in more uncertainty for the model around the ages at death of males. In females, the survival trajectory of this study is generally supported by previous estimates of life span in killer whales (Olesiuk et al., 1990, 2005). We used a Bayesian hierarchical approach to estimate the age‐specific survival, with the benefit of being able to include individuals with unknown age of birth and death. Through permutations, we were able to include individuals of unknown sex, which likely produces random variation in the model output. The southern resident data encapsulate a population that has near‐complete demographic data for all individuals compared with Northern resident and Bigg's killer whales where the data in older individuals in particular are sparser. This could affect the models’ ability to fit the maximum life span, potentially overestimating the life span, which is also evident in the larger variation in the age‐specific survival probability of Bigg's females (Figure 2c). However, the clear similarity in predicted life trajectories between especially the two resident populations (Figure 2) as well as the similarity between the modeled and observed survival probability (Figure S1) indicates that the model estimates are a good representation of the life trajectory of these three populations. To mitigate the increased uncertainty at maximum life span, we compared average (life expectancy at birth) and 90% longevity (age when 90% of the life span has occurred) (Figure 2) (Moorad et al., 2012; Ronget & Gaillard, 2020). This metric indicates that Bigg's females are expected to live longer than females from both resident populations with a difference of ~10 years (Figure 2). Despite the differences in the estimated life span, the results clearly show that all three populations have significant postreproductive periods with similar ages of onset of the postreproductive life stage in females (Table 5).

The cessation of female reproduction well before the end of life in a limited number of wild mammals largely remains an evolutionary puzzle. Yet, there is growing evidence that it has evolved based on the combined inclusive fitness benefits of helping and the costs of reproductive conflict for older females (Croft et al., 2015). The opportunity for females to help kin is integral to the adaptiveness of ceasing reproduction well before the end of life (Foster et al., 2012; Sear & Mace, 2008). Previous research in both humans and resident killer whales has shown that older females are able to provide help that positively affects the survival and reproduction of their kin (Foster et al., 2012; Hawkes et al., 1998; Lahdenperä et al., 2004). Especially in humans, offspring can have a long period after weaning, where they rely on older individuals for help providing food (Johnstone & Cant, 2010). Our study demonstrates that females of both resident and Bigg's killer whales can expect to live more than 20 years after they cease reproduction, allowing for a substantial period with the potential for helping kin. In resident killer whales, mothers impact the survival of their offspring well into their adult years (Foster et al., 2012), and grandmothers are important repositories for ecological knowledge for relatives (Brent et al., 2015) increasing the survival of their grandoffspring (Nattrass et al., 2019). Although dispersal of both males and females is more pronounced within the Bigg's ecotype, the postreproductive females are never observed on their own, but always with either their son(s) or daughter(s) (Towers et al., 2019). Further, the smaller matrilines may consist of up to three generations of maternally related kin and are regularly observed associating with individuals outside of the maternal group (Ford & Ellis, 1999; Towers et al., 2019). These patterns of association provide opportunities for social interactions that can lead to inclusive fitness benefits for older females (Brent et al., 2015; Towers et al., 2018). While the earlier dispersal of females from Bigg's groups likely reduces the occurrence of reproductive conflict between mothers and daughters (Figure 1) (Johnstone & Cant, 2010), Bigg's groups can also consist of reproductively active females from different generations, similar to the resident killer whales. The similarity in the timing of the cessation of reproduction of about 40 years in both the resident and Bigg's ecotypes indicates that Bigg's females may also experience intergenerational reproductive conflict and that this plays an important role in shaping fertility patterns across the different ecotypes. Testing this hypothesis requires more work on quantifying the kin structure and mortality patterns of Bigg's killer whales.

Here, we found that the differences in dispersal patterns between resident and Bigg's killer whales did not predict the timing of the cessation of reproduction and the length of the postreproductive life span. Similarly in humans, Snopkowski et al. (2014) compared age at menopause across ethnic groups with different patterns of postmarital residence to explore whether differences in dispersal pattern had an effect on the timing of menopause. They showed that female‐biased dispersal, expected to lead to an increase in female local relatedness with age, did not result in an earlier age at menopause compared with groups with male‐biased dispersal patterns (Snopkowski et al., 2014). It is possible that modern societies in both humans and killer whales are different from the ancestral societies in which the evolutionary effects of reproductive conflict and the timing of menopause occurred. The lack of variation in the timing of reproductive cessation despite differences in dispersal patterns has important implications for our understanding of the evolution of a long postreproductive period in mammals, including humans. Although patterns of kinship dynamics may predispose females to evolve a prolonged postreproductive life span, the costs of reproductive conflict and benefits helping are going to be shaped by the ecology of the population. For example, despite the close resemblance of demographic patterns between short‐ and long‐finned (*Globicephalas melas*) pilot whales (Alves et al., 2013; Augusto et al., 2017), indicating similar kinship patterns, a long postreproductive life span has only been observed in the short‐finned pilot whales (Ellis, Franks, Nattrass, Cant, Bradley, et al., 2017), which could be a result of difference in ecology between the two species. Moreover, there are several examples of primate species with female‐biased dispersal and local mating (e.g., chimpanzee, bonobos, and gorillas (Eriksson et al., 2006; Nishida et al., 2003; Stokes et al., 2003)), and thus an increase in female local relatedness with age similar to humans, where a prolonged postreproductive life span has not evolved (Ellis, Franks, Nattrass, Cant, Bradley, et al., 2017). It is likely that we may find similarly prolonged postreproductive life spans in other killer whale populations or other mammal species as we gather more individual‐based data on long‐lived social mammals. Some evidence already suggests that false killer whales (*Pseudorca crassidens*) (Photopoulou et al., 2017) and Asian elephants (*Elephas maximus*) have a long period as postreproductive, although for Asian elephants it is likely a social rather than physiological trait (Chapman et al., 2019).

Current models investigating the role of kinship dynamics for driving the evolution of menopause make specific predictions regarding how patterns of helping and harming will change with age (Cant & Johnstone, 2008; Johnstone & Cant, 2010) yet they do not predict how these patterns will drive the timing of menopause or the length of the postreproductive life span. Hence, our prediction that differences in dispersal patterns would lead to different selection pressures for a postreproductive period in the two different killer whale ecotypes is currently based on the reasoning that kinship dynamics are an indicator for the strength of selection for helping versus harming across the life span. The slightly earlier onset and longer expected postreproductive life span in Bigg's killer whales contrasts with our prediction that selection for a prolonged postreproductive period would be weaker in Bigg's compared with resident killer whales, based on the differences in kinship dynamics. Although these differences could arise from scarcer data on older females contributing to error associated with estimates of longevity, the results clearly show that Bigg's killer whales have a substantial postreproductive life span. This suggests that there could be substantial inclusive benefits of a postreproductive life span within this killer whale ecotype similar to the resident ecotype.

There is a striking similarity between our results and previous findings in humans of the timing (age) of menopause (e.g., Hazda hunter‐gatherer women: age *M* = 41 years and expected life span at age *M* = 26 years (Ellis, Franks, Nattrass, Cant, Bradley, et al., 2017)). Nonadaptive mechanisms associated with physiological constraints on the reproductive life span have been proposed as a driver for the evolution of menopause in mammals (Croft et al., 2015; Tully & Lambert, 2011; Wood et al., 2000). However, other long‐lived mammals, such as African elephants and baleen whales, breed for their entire life span (Mizroch, 1981; Moss, 2001) and accumulating evidence supports that adaptive benefits related to the postreproductive period for females in humans and killer whales are important drivers for this life history trait (Foster et al., 2012; Lahdenperä et al., 2004; Nattrass et al., 2019). It is possible that menopause cannot easily be reversed once evolved which may help explain the universality in the timing of menopause in humans, and likely also killer whales, despite the evident differences among societies, such as patterns of dispersal (Snopkowski et al., 2014).

## 5 CONCLUSION

In conclusion, when taken together with previous work, our findings support the hypothesis that kinship dynamics play a key role in the evolution of a prolonged postreproductive life span in killer whales. However, contrary to our predictions, the timing and expected duration of the postreproductive life span did not vary with the dispersal pattern from the natal group, which likely represents different costs and benefits of helping and harming in the two ecotypes. These were, however, not taken into account in our predictions, but would be valuable to disentangle in future research to better understand the drivers for the timing of long postreproductive life spans in mammals. Nevertheless, the clear similarity of the postreproductive period in some of the most genetically distinct killer whale populations echoes what has been observed across different human societies. This indicates a long postreproductive period being an ancestral trait in killer whales, and likely present in other killer whale populations and ecotypes.

## CONFLICT OF INTEREST

The authors declare that there are no competing interests.

## AUTHOR CONTRIBUTION

**Mia Lybkær Kronborg Nielsen:** Conceptualization (equal); Formal analysis (lead); Methodology (equal); Visualization (lead); Writing‐original draft (lead); Writing‐review & editing (lead). **Samuel Ellis:** Conceptualization (equal); Formal analysis (supporting); Methodology (equal); Supervision (equal); Writing‐review & editing (supporting). **Jared R. Towers:** Data curation (lead); Funding acquisition (equal); Writing‐review & editing (supporting). **Thomas Doniol‐Valcroze:** Data curation (equal); Formal analysis (supporting); Funding acquisition (equal); Resources (equal); Writing‐review & editing (supporting). **Daniel Franks:** Conceptualization (equal); Formal analysis (supporting); Methodology (supporting); Writing‐review & editing (supporting). **Michael Cant:** Conceptualization (equal); Formal analysis (equal); Funding acquisition (equal); Methodology (equal); Supervision (equal); Writing‐review & editing (supporting). **Michael Weiss:** Data curation (equal); Formal analysis (supporting); Writing‐review & editing (supporting). **Rufus A. Johnstone:** Formal analysis (equal); Methodology (equal); Writing‐review & editing (supporting). **Kenneth C. Balcomb III:** Data curation (lead); Funding acquisition (equal); Writing‐review & editing (supporting). **David K. Ellifrit:** Data curation (lead); Funding acquisition (equal); Writing‐review & editing (supporting). **Darren P. Croft:** Conceptualization (lead); Formal analysis (supporting); Funding acquisition (lead); Methodology (equal); Project administration (lead); Supervision (lead); Writing‐review & editing (supporting).

## Supporting information

Supplementary Material

## Data Availability

Data to replicate the analyses are available from the online repository: https://doi.org/10.5061/dryad.6t1g1jwxx. Further enqcuiries about the data can be directed to the authors, the Center for Whale Research (https://www.whaleresearch.com), or Fisheries and Oceans Canada (https://www.dfo‐mpo.gc.ca).
