# Supplementary material for: A long postreproductive life span is a shared trait among genetically distinct killer whale populations
Source: Ecol Evol. 2021 Jun 16;11(13):9123–36. doi: 10.1002/ece3.7756 (PMC8258204; doi:10.1002/ece3.7756)
Supplement: Supplementary file 1 — Supplementary Material [file ECE3-11-9123-s001.docx]

# Supplementary materials for

# **A long post-reproductive lifespan is a shared trait among genetically distinct killer whale populations**

**This PDF file includes:**

Supplementary methods

Figure S1

Supplementary methods:

The observed survival probability is calculated using the Kaplan-Meier survival model (package *survival* in R). To be able to investigate the survival probability with juvenile mortality included, we randomly assigned sex to individuals of unknown sex. Only individuals of known ages are used to calculate the observed survival probability, meaning that survival is censored at the length of each study period. The observed survival probability is plotted together with the summarised survival trajectory of all 1000 permutations for each population.


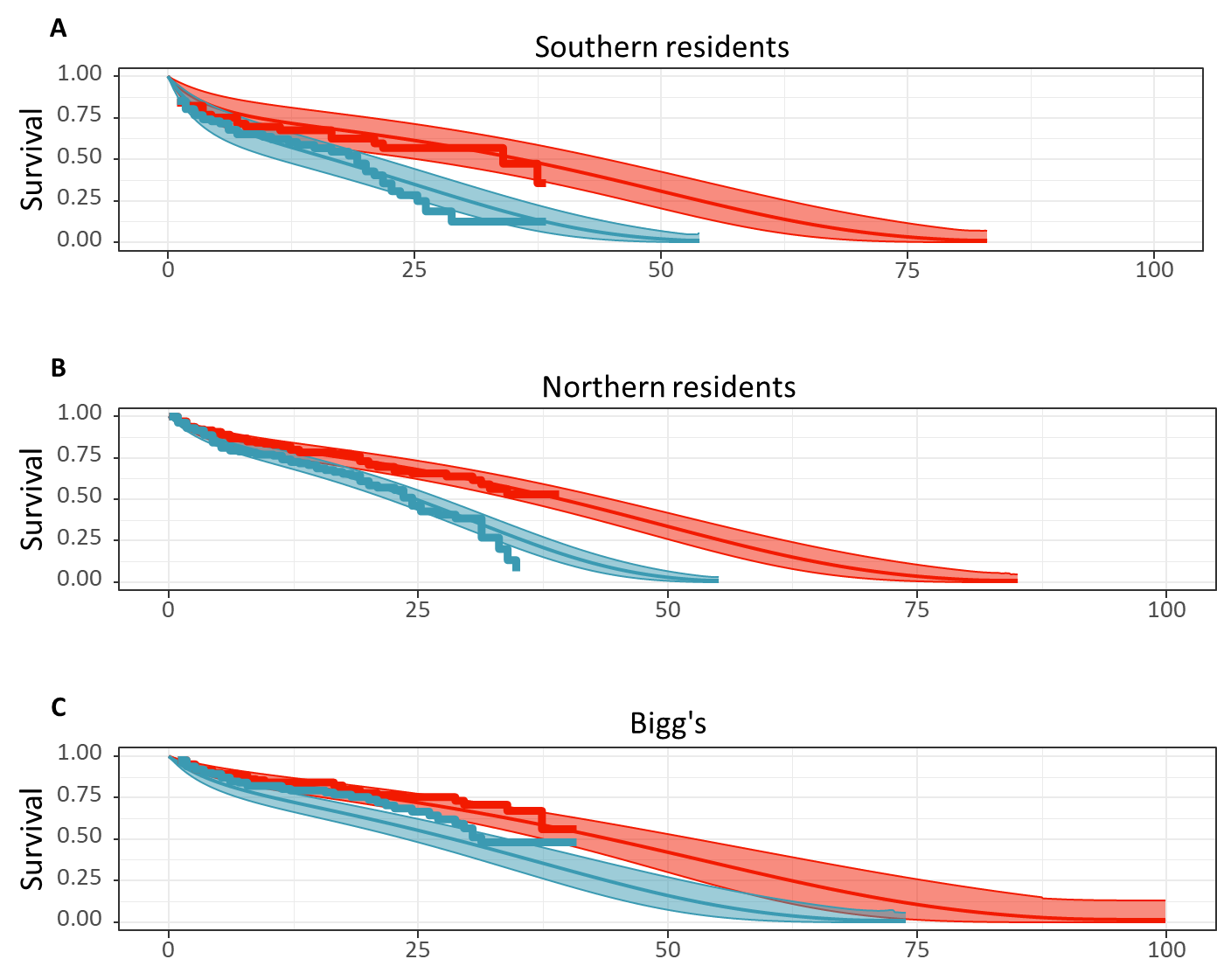


Figure S1: Observed and estimated survival probability plotted together for all three killer whale populations. Red is females and blue is males. The smooth line and shaded areas represent the summarised median and 95% credible intervals, respectively, for all 1000 permutations. The bold, step-wise line represent the observed survival probability from a Kaplan-Meier survival model.
